# Analysis of conserved microRNAs in floral tissues of sexual and apomictic *Boechera *species

**DOI:** 10.1186/1471-2164-12-500

**Published:** 2011-10-11

**Authors:** Samuel Amiteye, José M Corral, Heiko Vogel, Timothy F Sharbel

**Affiliations:** 1 Leibniz Institute of Plant Genetics and Crop Plant Research (IPK), Corrensstrasse 3, D-06466 Gatersleben, Germany; 2 Max Planck Institute for Chemical Ecology, Department of Entomology, Genomics Research Group, Hans-Knöll-Strasse 8, D-07745 Jena, Germany

## Abstract

**Background:**

Apomixis or asexual seed formation represents a potentially important agronomic trait whose introduction into crop plants could be an effective way to fix and perpetuate a desirable genotype through successive seed generations. However, the gene regulatory pathways underlying apomixis remain unknown. In particular, the potential function of microRNAs, which are known to play crucial roles in many aspects of plant growth and development, remains to be determined with regards to the switch from sexual to apomictic reproduction.

**Results:**

Using bioinformatics and microarray validation procedures, 51 miRNA families conserved among angiosperms were identified in *Boechera*. Microarray assay confirmed 15 of the miRNA families that were identified by bioinformatics techniques. 30 cDNA sequences representing 26 miRNAs could fold back into stable pre-miRNAs. 19 of these pre-miRNAs had miRNAs with *Boechera*-specific nucleotide substitutions (NSs). Analysis of the Gibbs free energy (ΔG) of these pre-miRNA stem-loops with NSs showed that the *Boechera*-specific miRNA NSs significantly (p ≤ 0.05) enhance the stability of stem-loops. Furthermore, six transcription factors, the Squamosa promoter binding protein like SPL6, SPL11 and SPL15, Myb domain protein 120 (MYB120), RELATED TO AP2.7 DNA binding (RAP2.7, TOE1 RAP2.7) and TCP family transcription factor 10 (TCP10) were found to be expressed in sexual or apomictic ovules. However, only SPL11 showed differential expression with significant (p ≤ 0.05) up-regulation at the megaspore mother cell (MMC) stage of ovule development in apomictic genotypes.

**Conclusions:**

This study constitutes the first extensive insight into the conservation and expression of microRNAs in *Boechera *sexual and apomictic species. The miR156/157 target squamosa promoter binding protein-like 11 (SPL11) was found differentially expressed with significant (p ≤ 0.05) up-regulation at the MMC stage of ovule development in apomictic genotypes. The results also demonstrate that nucleotide changes in mature miRNAs significantly (p ≤ 0.05) enhance the thermodynamic stability of pre-miRNA stem-loops.

## Background

Apomixis, or asexual reproduction through seeds, is a naturally occurring reproductive form which has been observed in more than 400 plant species. Apomictic reproduction is, however, absent in many agriculturally important crop plants [1]. It therefore represents a potentially important agricultural tool, since introduction of apomixis into crops could be an effective way to fix and propagate a given genotype for superior crop performance. Apomixis has evolved from many different sexual taxa [2,3], although the genetic factors underlying apomictic reproduction remain unknown.

The genus *Boechera *(Bocher's rock cress; formerly *Arabis*) is monophyletic, has a basic chromosome number × = 7 [4], and wild populations are characterized by diploid sexuals, and diploid, aneuploid, and polyploid (mostly 2n = 3x = 21) apomicts [5]. Plants of this genus are perennial members of the Brassicaceae which are distributed throughout North America and Greenland [4,6,7]. The switch from sexual to apomictic reproduction has been hypothesized to arise via de-regulation of the developmental pathways originally leading to sexual seed formation [8]. As virtually all asexual plants or animals are hybrid and/or polyploid, their associated gene regulatory changes have been proposed as possible triggers for the switch in reproductive mode [9]. In particular, the potential function of microRNAs (miRNAs), which are known to play crucial roles in many aspects of plant development, remains to be determined with regards to the switch from sex to apomixis.

MiRNAs are 20-24 nucleotide small endogenous non-protein-coding regulatory RNA sequences that are produced by genes distinct from the genes that they regulate. Evidence provided by Allen et al [10] and Felippes et al [11] show that some miRNAs evolved by inverted duplications of target gene sequences, whereas others originated from random sequences that either have self-complementarity by chance or sequences that represent highly eroded inverted duplications. Since their discovery, several miRNAs have been computationally and/or experimentally identified and characterized in different species. A number of studies have shown that miRNAs play key roles in regulatory functions of gene expression for most eukaryotes [12,13], mainly at the post-transcriptional levels [14,15]. Several recent findings have implicated miRNAs in a number of biological mechanisms including leaf [16], stem [15] and root growth [17], floral organ identity, control of female gamete formation and reproductive development [18,19], auxin signaling [20], and biotic and abiotic stress response [13].

Biogenesis of miRNAs involves nucleolytic processing of a precursor transcript with extensive foldback structure [21-23]. miRNAs are initially expressed as part of longer transcripts that are self-complementary foldback hairpin structures termed primary miRNAs (pri-miRNAs). Pri-miRNA precursors are transcribed by miRNA genes which are mostly independent transcript units. These pri-miRNA precursors are first processed into pre-miRNAs from which miRNAs are eventually generated by the ribonuclease III nucleases and Dicer-like1 (DCL1) in plants. Subsequently, the mature single stranded miRNA is incorporated into a miRNA-induced silencing complex (miRISC) to cleave its specific target messenger RNA (mRNA), or to effect translational attenuation of its target transcript [24,25]. Plant miRNAs bind to the protein-coding region of their target mRNAs to induce target mRNA degradation via an RNAi-like mechanism where an Argonaut (AGO) protein cleaves the miRNA-mRNA duplex, thereby repressing expression of that particular mRNA [26]. It is also known that gene repression can be effected by translational inhibition through deadenylation of the 3' poly (A) tail and decapping of the 5' end in mRNAs, which leads to progressive mRNA decay and degradation [27,28].

Accurate detection and expression profiling of miRNAs will enable a better understanding of their role in plant growth and development [13,18,20], and could provide insights into miRNA-mediated apomictic gene regulatory mechanisms. The main approaches for miRNA identification have been widely undertaken by computational prediction, direct cloning and sequencing. Until recently, most sequence information including *Expressed Sequence Tags *(ESTs) or *Genome Survey Sequences *(GSS) used for computational prediction of miRNAs were generated by traditional Sanger sequencing methods [29,30]. Compared to highly conserved miRNAs, less- or non-conserved miRNAs are often expressed at lower levels, thus making their detection more daunting using small-scale sequencing. The development of next generation sequencing technology has greatly improved the capacity to identify low abundance or tissue-specific miRNAs, and has enhanced the discovery of several conserved, non-conserved or lowly expressed miRNAs through cloning and deep sequencing of small RNA and transcriptome libraries in *Arabidopsis thaliana *[31,32], *Triticum aestivum *(wheat; [33]), *Solanum lycopersicum *(tomato [34]), *Oryza sativa *(rice), *Populus trichocarpa *(cotton wood), and *Manihot esculenta *(Cassava) [35-37]. To date, many varieties of miRNAs are reported in plants, animals, and even microbes [38].

Although miRNAs have been studied in plants for years, no extensive study has yet been performed on *Boechera*. The objective of this work was thus to identify and completely catalogue conserved plant miRNAs, and to compare the expression pattern of their target genes in the floral tissues of sexual and apomictic *Boechera*, in order to shed light on the potential role of miRNAs in the switch from sexual to apomictic reproduction. To do so we have cloned, sequenced and validated conserved miRNAs using bioinformatics and microarray techniques, and have analyzed these data using sexual and apomictic EST libraries (sequenced using 454 FLX technology) and comparative expression profiles between microdissected ovules from sexual and apomictic genotypes [39,40].

## Results and Discussion

### Homology of miRNAs to *Boechera *ESTs

**Figure 1:**
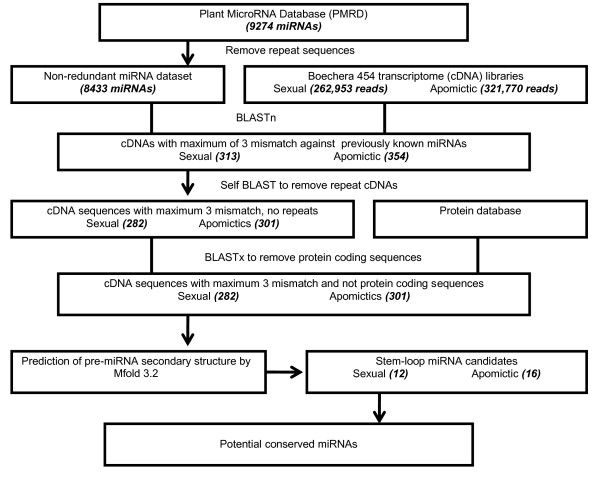
**Scheme for search of conserved miRNAs in *Boechera *species**.

The BLASTn search using a reference set of 8433 non-redundant known conserved plant miRNAs against flower-specific sexual and apomictic *Boechera *EST libraries led to the identification of 282 sexual and 301 apomictic transcripts with high homology to miRNAs of other plant species (Figure 1). Of these, 13 sexual and 16 apomictic transcripts could fold back into stable hairpins containing conserved miRNAs (Table 1 &2; Additional file 1, Figure S1). Many EST sequences were found that could not fold back into stem loops, although it is unclear whether this was due to the fact that they were not pre-miRNAs or whether this was due to sequencing errors introduced by the 454 FLX system. Predominantly, the less conserved miRNA families (e.g. miR444 to miR869) matched a small number of cDNA sequences which in most cases were found to be truncated precursor sequences in the EST libraries, and thus could not fold into stable stem-loops (Table 1 &2).

**Table 1:** Characteristics of conserved miRNA families and stem-loops in sexual *Boechera *genotypes.

miRNA family	Mature miRNAs	Plant sp., NSs	NN	ARM	A+U%	AMFE	MFEI	EST ID	**EMBL No**.
miR156	UGACAGAAGAGAG***A***GAGCAC	Ath, **U**/A	75	5'	54.67	28.80	0.635	ET5PU7E02HBOCM	FR869734
miR157	UUGACAGAAGAGAG***A***GAGCAC	Sbi, **U**/A	75	5'	54.67	28.80	0.635	ET5PU7E02HBOCM	FR869734
miR159	UUUGG***U***UUGAAGG***A***AGCUCUA	Ath, **A**/U, **G**/A	-	-	-	-	-	ETM6Q5C04I3XE2**^a^**	FR869757
miR160	UGCCUGGCUCCCUGUAUGCCA	Ath	-	-	-	-	-	ET5PU7E02JH8DI**^a^**	FR869730
miR161	UGAAAGUGACUACAUCGGGGU	Ath	92	5'	55.43	24.67	0.554	ET5PU7E02IM9YA	FR869722
miR164	UGGAGAAGCAGGGCACGUAAA	Gar	-	-	-	-	-	ETM6Q5C04IAM5V**^a^**	FR869752
miR166	CCGGACCAGGCUUCAUCCCAG	Pta	-	-	-	-	-	ET5PU7E02JKLSI**^a^**	FR869725
miR167	UGAAGCUGCCAGCAUGAUCUA	Ath	100	5'	60.00	48.20	1.201	ETM6Q5C03GWWM2	FR869745
miR170	UGAUUGAGCCGCGCCAAUAUC	Ath	-	-	-	-	-	ETM6Q5C03GVN0G**^a^**	FR869746
miR172	AGAAUC***C***UGAUGAUGCUGCAU	Ath, **U**/C	-	-	-	-	-	ET5PU7E02GZHSB**^a^**	FR869721
miR319	UUGGA***A***UGAAGGGAGCUCC***AC***	Ath, **A**/C, **U**/A, **U**/C	-	-	-	-	-	ETM6Q5C03FYJ8Y**^a^**	FR869740
miR394	UUGGCAUUCUGUCCACCUCC	Ath	116	5'	57.76	46.46	1.100	ET5PU7E02IYKJ5	FR869733
miR395	AUGAAG***A***GUUUGGAGGAACUC	Osa, **U**/A	-	-	-	-	-	ETM6Q5C03FTE98**^a^**	FR869741
miR396	UCCACAGGCUUUCUUGAACGG	Ghr	143	5'	41.96	38.37	0.661	ET5PU7E02GM4DS	FR869731
miR398	UGUG***A***UCUCAGGU***A***ACCCCUU	Ath, **U**/A, **C**/A	-	-	-	-	-	ETM6Q5C04IDRCB**^a^**	FR869755
miR399	UGCCAAAGGAGAU***A***UGCCCU***A***	Ath, **U**/A, **G**/A	-	-	-	-	-	ET5PU7E02FZ073**^a^**	FR869726
miR400	UAUGAGAGUAUUAUA***U***GUCAC	Ath, **A**/U	76	3'	60.53	15.79	0.400	ET5PU7E02JJRIA	FR869735
miR403	UUAGAUUCACGCACAAACUC***C***	Ath, **G**/C	75	5'	57.33	24.93	0.584	ET5PU7E02I3RXE	FR869723
miR408	AUGCACUGCCUCUUCCCUGGC	Ath	148	3'	58.78	33.58	0.815	ETM6Q5C04JX15C	FR869749
miR414	UCAUC***A***UCAUCAUCAUCGUC***G*** UCAUC***A***UCAUCAUCAUCGUCA UCAUC***A***UCAUCAUCAUCGUCA	Ath, **U**/A, **A**/G Ath, **U**/A Ath, **U**/A	170 221 233	5' 3' 3'	51.18 51.01 56.65	29.29 23.62 24.64	0.600 0.482 0.568	ET5PU7E02IZWR4 ET5PU7E02GNU3F ET5PU7E02I14IE	FR869738 FR869727 FR869724
miR415	***G***ACAGAG***A***AGAAACAGAACAU	Ath, **A**/G, **C**/A	-	-	-	-	-	ETM6Q5C03FIL8C**^a^**	FR869748
miR444	UUGCUGCCUCAAGCU***C***CC***G***GC	Zma, **U**/C, **U**/G	-	-	-	-	-	ETM6Q5C04IXD3L**^a ^**	FR869750
miR482	UCUUCCCUACACC***G***CCCAUAC	Gso, **U**/G	-	-	-	-	-	ET5PU7E02HNZGI**^a^**	FR869720
miR529	***G***CU***C***U***U***CCCUCUCUCUUCUUC	Osa, **C**/G, **G**/C, **A**/U	-	-	-	-	-	ET5PU7E02HC551**^a^**	FR869729
miR824	UAGACCAUUUGUGAGAAG***A***GA	Ath, **G**/A	-	-	-	-	-	ETM6Q5C04ISA8K**^a^**	FR869754
miR835	UU***U***UU***C***CAUAUGUUCUUUAUC	Ath, **C**/U, **G**/C	-	-	-	-	-	ETM6Q5C04JNEVJ**^a^**	FR869751
miR838	UUUUCUUCUACUUCUU***C***C***C***CA	Ath, **G**/C, **A**/C	-	-	-	-	-	ETM6Q5C03FOEE6**^a^**	FR869747
miR841	UACGA***C***CCACU***G***GAAACUGAA	Ath, **G**/C, **U**/G	-	-	-	-	-	ETM6Q5C03HCEIS**^a^**	FR869742
miR845	UAGCUCUGAUACCAA***A***UGAUA	Vvi, **U**/A	-	-	-	-	-	ET5PU7E02F58WO**^a^**	FR869732
miR846	UUGAAUUG***G***AGUGCUUG***C***AUU	Ath, **A**/G, **A**/C	-	-	-	-	-	ETM6Q5C03FVWRY**^a^**	FR869743
miR852	AAGAUAAGCGCCUUAG***G***UCUG	Ath, **U**/G	89	5'	62.92	38.31	1.033	ETM6Q5C03G2GJO	FR869744
miR854	GAUGAGGA***G***A***A***GGAGGAGGAG	Ath, **U**/G, **G**/A	-	-	-	-	-	ETM6Q5C04JC8OP**^a^**	FR869756
miR859	UCUCUCUGUUGUGAA***A***UCAAA	Ath, **G**/A	-	-	-	-	-	ET5PU7E02GYEM5**^a^**	FR869736
miR860	UCA***G***UAG***C***UUGGACUAUGUAU	Ath, **A**/G, **A**/C	-	-	-	-	-	ETM6Q5C03G8ZLD**^a^**	FR869739
miR861	CCUUGGAGAAAUAUGC***U***UCAA	Ath, **G**/U	-	-	-	-	-	ET5PU7E02IMVAL**^a^**	FR869728
miR865	UUU***C***UCCUCAAAUUU***C***UCCAA	Ath, **U**/C, **A**/C	-	-	-	-	-	ETM6Q5C04JQMWD**^a^**	FR869753
miR869	CAUGGUUCAAUGC***A***GGUG***C***UA	Gma, **U**/A, **U**/C	-	-	-	-	-	ET5PU7E02JV51F**^a^**	FR869737

**Plant sp, NSs**, Nucleotide substitutions between known plant query miRNAs and the corresponding miRNA in *Boechera *sexual species; **NN**, Number of nucleotides hairpin length; **ARM**, mature miRNA location in hairpin structure; **AMFE**, Adjusted minimum fold energy; **MFEI**, Minimum fold energy index; **EST ID**, Identifier of the 454 transcripts from which miRNA was derived. Italicized, bold and underlined red letters show nucleotide substitutions in miRNAs of *Boechera *sexual species. **^a^**EST could not form secondary stem-loop structures. **EMBL No.**, European Molecular Biology Laboratory accession number; **Plant species: Ath**, *Arabidopsis thaliana; ***Gar**, *Gossypium arboreum*; **Ghr**, *Gossypium hirsutum; ***Gma**, *Glycine max; ***Gso**, *Glycine soja; ***Osa**, *Oryza sativa; ***Pta**, *Pinus taeda; ***Sbi**, *Sorghum bicolor; ***Vvi**, *Vitis vinifera; ***Zma**, *Zea mays*.

**Table 2:** Characteristics of conserved miRNA families and stem-loops in apomictic *Boechera *genotypes.

miRNA family	Mature miRNAs	Plant sp., NSs	NN	ARM	A+U%	AMFE	MFEI	EST ID	**EMBL No**.
miR156	UGACAGAAGAGAG***A***GAGCAC UGACAGAAGAGAG***A***GAGCAC	Ath, **U**/A Ath, **U**/A	66 105	5' 5'	53.03 52.38	30.00 25.24	0.639 0.530	ETM6Q5C01AY29E ET5PU7E01BE5BP	FR869781 FR869766
miR157	UUGACAGAAGAGAGAG***G***GCAC	Ath, **A**/G	119	5'	57.98	32.10	0.764	ET5PU7E01A5S8V	FR869768
miR159	UUUGGA***C***UGAAGGGAGCUCCU	Ath, **U**/C	-	-	-	-	-	ETM6Q5C02EBWUA**^a^**	FR869788
miR160	UGCCUGGCUCCCUGUAUGCCA	Ath	110	5'	58.18	42.10	1.007	ET5PU7E01AQT2A	FR869776
miR161	UCAAUGCAUUGAAAGU***A***ACUA	Ath, **G**/A	-	-	-	-	-	ETM6Q5C01AMWRE**^a^**	FR869779
miR162	UCGAUAAACCUCUGCAUCCAG	Ptc	84	3'	55.95	48.45	1.100	ETM6Q5C01AZ87O	FR869778
miR166	CCGGACCAGGCUUCAUCCC***CC***	Pta, **A**/C, **G**/C	-	-	-	-	-	ET5PU7E01CXVM2**^a^**	FR869765
miR167	UGAAGCUGCCAGCAUGAUCUA	Ath	100	5'	60.00	48.20	1.205	ETM6Q5C01CA126	FR869786
miR169	UGAGCCAAGGAUGA***U***UUGCC***U***	Ath, **C**/U, **G**/U	-	-	-	-	-	ETM6Q5C01B8RXC**^a^**	FR869787
miR170	UGAUUGAGCCGCGCCAAUAUC	Ath	121	3'	51.24	40.50	0.831	ET5PU7E01EN973	FR869761
miR172	AGAAUCUUGAUGAUGCUGCAU	Ath	142	3'	49.30	22.39	0.442	ET5PU7E01CV6Q5	FR869764
miR319	UUGGACUGAAGGGAGCUCCUU	Ath	184	3'	57.61	45.20	1.066	ETM6Q5C02EBWUA	FR869788
miR394	UUGGCAUUCUGUC***A***ACCUCC	Ath, **C**/A	126	3'	57.94	19.13	0.455	ET5PU7E01CBSOI	FR869772
miR395	AUGAAG***A***GUUUGGAGGAACUC	Osa, **U**/A	-	-	-	-	-	ETM6Q5C02DVQZ4**^a^**	FR869794
miR396	UCCACAGGCUUUCUUGAACGG	Ghr	-	-	-	-	-	ETM6Q5C02DSTK1**^a^**	FR869791
miR398	UGUGUUCUCAGGUCACCCCUU	Ath	-	-	-	-	-	ET5PU7E01B8LVW**^a^**	FR869770
miR400	UAUGAGAGUAUUAUA***G***GUCAC	Ath, **A**/G	-	-	-	-	-	ET5PU7E01AVMRY**^a^**	FR869771
miR408	AUGCACUGCCUCUUCCCUGGC	Ath	89	3'	52.81	39.55	0.838	ET5PU7E01EE6T6	FR869769
miR414	UCAUC***A***UCAUCAUCAUCGUC***U***	Ath, **U**/A, **A**/U	208	3'	57.69	16.92	0.400	ET5PU7E01DL36L	FR869767
	UCAUC***A***UCAUCAUCAUCGUC***G***	Ath, **U**/A, **A**/G	170	5'	50.88	29.29	0.603	ET5PU7E01BXM22	FR869762
	UCAUC***G***UCAUCAUCAUCGUCA	Ath, **U**/G	104	5'	63.46	29.33	0.803	ET5PU7E01D5L0P	FR869759
miR415	***G***ACAGAG***A***AGAAACAGAACAU	Ath, **A**/G, **C**/A	135	5'	56.30	24.96	0.571	ETM6Q5C01A4TW0	FR869780
miR472	UUUU***G***CCUACUCC***A***CCCAUACC	Ath, **U**/G, **G**/A	-	-	-	-	-	ETM6Q5C01B63E7**^a^**	FR869782
miR529	CU***C***U***U***CCCUCUCUCUUCUUC	Osa, **G**/C, **A**/U	-	-	-	-	-	ETM6Q5C02D6DWQ**^a^**	FR869795
miR776	UCUAA***U***UCUUCUAUUGAU***A***UU	Ath, **G**/U, **G**/A	-	-	-	-	-	ET5PU7E01CU8R6**^a^**	FR869774
miR820	UCG***UA***CUCGUGGAUGGACCAG	Osa, **G**/U, **C**/A	-	-	-	-	-	ET5PU7E01CXV4L**^a^**	FR869760
miR824	UAGACCAUUUGUGAGAAG***A***GA	Ath, **G**/A	-	-	-	-	-	ETM6Q5C01BUUMV**^a^**	FR869777
miR835	UU***U***UU***C***CAUAUGUUCUUUAUC	Ath, **C**/U, **G**/C	-	-	-	-	-	ET5PU7E01BKF2J**^a^**	FR869773
miR840	ACACUGAAGGA***G***CU***G***AACUAA***U***	Ath, **C**/G, **A**/G. **C**/U	-	-	-	-	-	ETM6Q5C02C26W5**^a^**	FR869789
miR841	UACGA***C***CCACU***G***GAAACUGAA	Ath, **G**/C, **U**/G	-	-	-	-	-	ETM6Q5C01B0IVV**^a^**	FR869785
miR846	UUGAAUUG***G***AGUGCUUG***C***AUU	Ath, **A**/G, **A**/C	-	-	-	-	-	ETM6Q5C02D2FL9**^a^**	FR869793
miR854	GAUGA***U***GAUAG***U***GAGGAGGAG	Ath, **G**/U, **G**/U	-	-	-	-	-	ETM6Q5C01A9E26**^a^**	FR869783
miR857	UU***A***UGUAUGUUGAA***U***GUGUAU	Ath, **U**/A, **G**/U	-	-	-	-	-	ETM6Q5C01AWYYJ	FR869784
miR859	UCUCUCUGUUGUGAA***A***UCAAA	Ath, **G**/A	-	-	-	-	-	ET5PU7E01E1HDI**^a ^**	FR869775
miR860	UCA***G***UAG***C***UUGGACUAUGUAU	Ath, **A**/G, **A**/C	-	-	-	-	-	ETM6Q5C02DJ9H7**^a^**	FR869790
miR861	CCUUGGAGAAAU***G***UGC***U***UCAA	Ath, **A**/G, **G**/U	233	5'	51.93	31.93	0.664	ET5PU7E01A7VRK	FR869763
miR865	UUU***C***UCCUCAAAUUU***C***UCCAA	Ath, **U**/C, **A**/C	-	-	-	-	-	ETM6Q5C02DTC6W**^a^**	FR869792
miR869	CAUGGUUCAAUGC***A***GGUGUUA	Gma, **U**/A	-	-	-	-	-	ET5PU7E01A48FH**^a^**	FR869758

**Plant sp, NSs**, Nucleotide substitutions between known plant query miRNAs and the corresponding miRNA in *Boechera *apomictic species; **NN**, Number of nucleotides hairpin length; **ARM**, mature miRNA location in hairpin structure; **AMFE**, Adjusted minimum fold energy; **MFEI**, Minimum fold energy index; **EST ID**, Identifier of the 454 transcripts from which miRNA was derived. Italicized, bold and underlined red letters show nucleotide substitutions in miRNAs of *Boechera *apomictic species. **^a^**EST could not form secondary stem-loop structures. **EMBL No.**, European Molecular Biology Laboratory accession number; **Plant species: Ath**, *Arabidopsis thaliana; ***Ghr**, *Gossypium hirsutum; ***Gma**, *Glycine max; ***Osa**, *Oryza sativa; ***Pta**, *Pinus taeda; ***Ptc**, *Populus trichocarpa; ***Sbi**, *Sorghum bicolor*.

### Bioinformatically-identified conserved miRNA families

**Figure 2:**
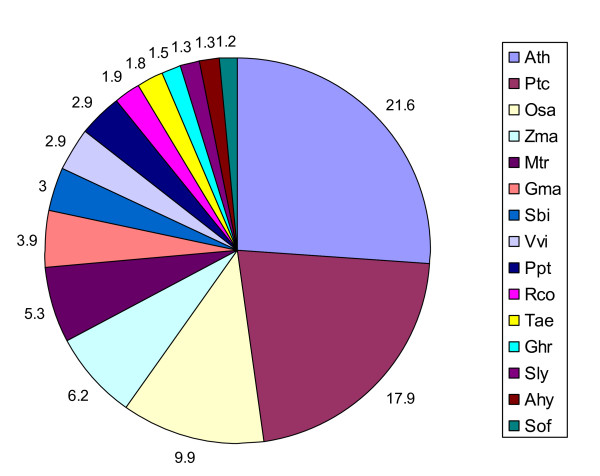
**Percentage of conserved miRNAs between *Boechera *and other plant species (species with < 1% not included)**. Ath, *Arabidopsis thaliana; *Ptc, *Populus trichocarpa; *Osa, *Oryza sativa; *Zma, *Zea mays; *Mtr, *Medicago truncatula; *Gma, *Glycine max; *Sbi, *Sorghum bicolor; *Vvi, *Vitis vinifera; *Ppt, *Physcomitrella patens; *Rco, *Ricinus communis; *Tae, *Triticum aestivum; *Ghr, *Gossypium hirsutum; *Sly, *Solanum lycopersicum; *Ahy, *Arachis hypogaea; *Sof, *Saccharum officinarum*.

**Figure 3:**
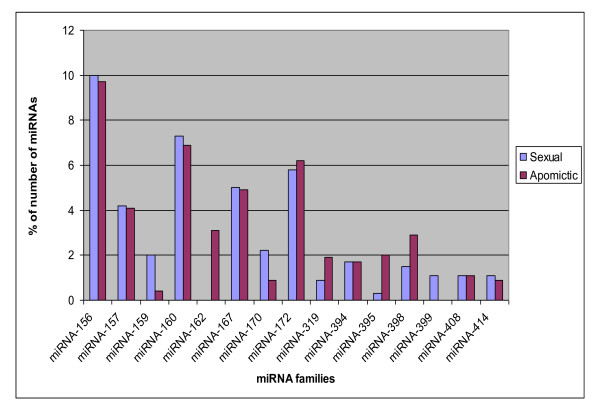
**Abundance (based on total number of transcripts) of conserved miRNAs in *Boechera *species**.

**Figure 4:**
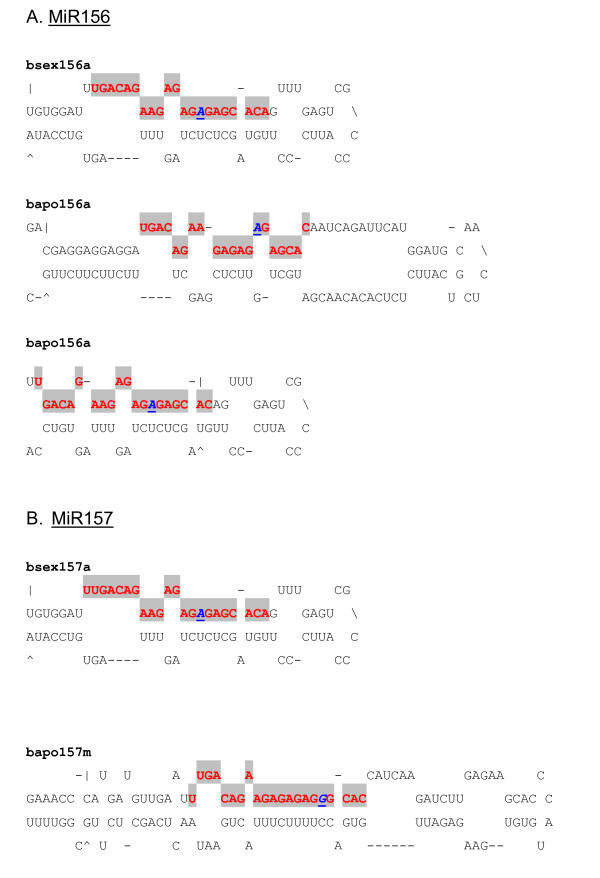
**Predicted pre-miRNA stem-loops of miR156/157 families with nucleotide substitutions in *Boechera *species**. Shaded red letters correspond to the sequence of the mature miRNA. Nucleotide substitutions of conserved miRNAs in other plant species compared with the corresponding miRNAs in *Boechera *species are shown as italicized, bold and underlined blue letters. MiRNA precursors could be slightly longer than the sequences shown in this figure.

**Figure 5:**
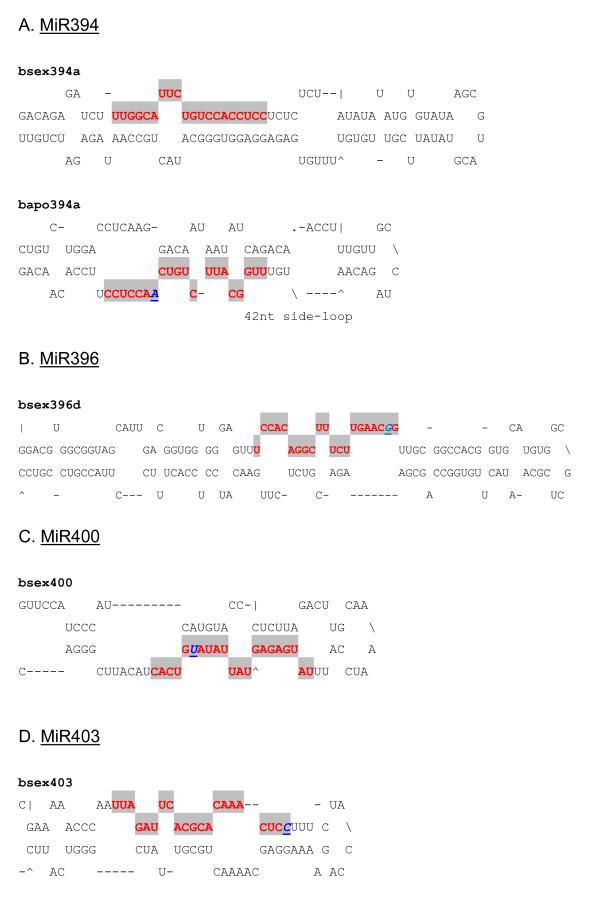
**Predicted pre-miRNA stem-loops of miRNA families with nucleotide substitutions in *Boechera *species**. Shaded red letters correspond to the sequence of the mature miRNA. Nucleotide substitutions of conserved miRNAs in other plant species compared with the corresponding miRNAs in *Boechera *species are shown as italicized, bold and underlined blue letters. MiRNA precursors could be slightly longer than the sequences shown in this figure.

In all, 44 miRNA families across 67 plant species were found to match at least one *Boechera *454 EST read, with *A. thaliana *being the predominant species (Figure 2). Conserved plant miRNA families in *Boechera *were identified to a large extent based upon high homology with reported conserved *A. thaliana *miRNAs (Figure 2). In cases where *Boechera *and *A. thaliana *did not share particular miRNA families, a search for conserved miRNA families was performed in other plant species. The predominant miRNA families which shared similarity with the highest number of *Boechera *454 reads were miR156, miR157, miR160, miR167 and miR172 (Figure 3). It was observed also that the *Boechera *miRNAs exhibit a wide variation in the length of pre-miRNA sequences (Table 1 &2; Figure 4, 5, 6 &7). 29 families were found to be common between the sexual and apomictic genotypes. Of these, 17 mature miRNAs (miR156, 160, 167, 170, 172, 395, 396, 408, 415, 529, 824, 835, 841, 846, 859, 860 and 865) were similar in sequence, whereas 12 were different in sequence constitution due to nucleotide differences between the two reproductive modes. These included miRNAs miR157, 159, 161,166, 319, 394, 398, 400, 414, 854, 861 and 869 (Table 1 &2). Pre-miRNA lengths varied from 66 to 233 nucleotides, with most between 66 and 184 nucleotides, a length similar to that of pre-miRNAs in other species. The location of the mature miRNAs in the precursor pre-miRNAs also varied among the miRNA families. In 12 pre-miRNAs, the miRNAs were found in the 3' arm while 18 were in the 5' arm of the stem-loop hairpin structures (Table 1 &2; Figure 4, 5, 6 &7).

**Figure 6:**
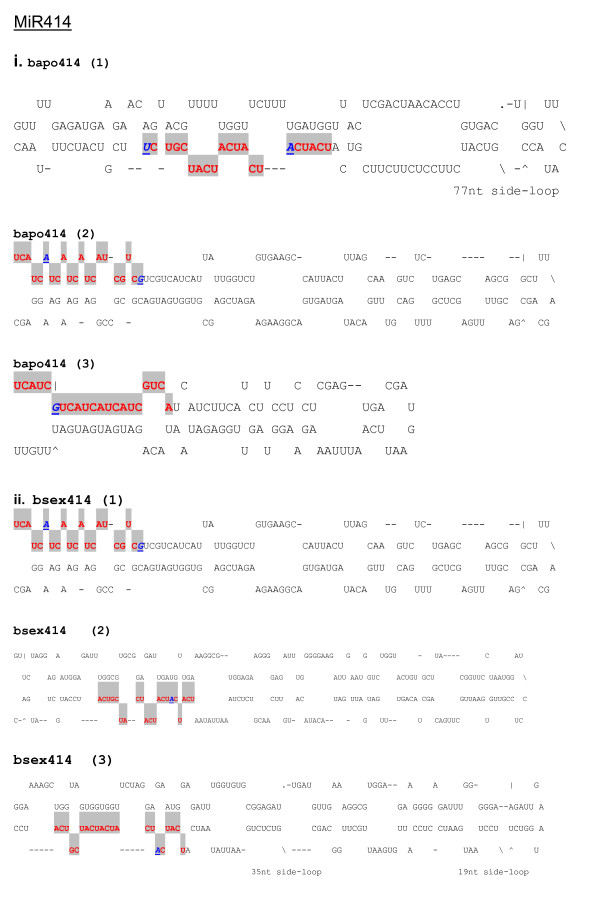
**Predicted pre-miRNA stem-loops of miR414 family with nucleotide substitutions in *Boechera *species**. Shaded red letters correspond to the sequence of the mature miRNA. Nucleotide substitutions of conserved miRNAs in other plant species compared with the corresponding miRNAs in *Boechera *species are shown as italicized, bold and underlined blue letters. MiRNA precursors could be slightly longer than the sequences shown in this figure.

**Figure 7:**
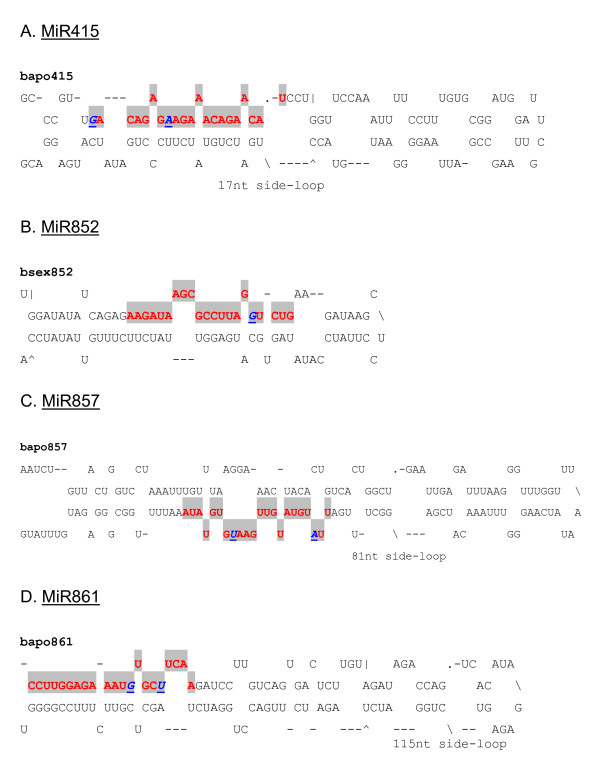
**Predicted pre-miRNA stem-loops of miRNA families with nucleotide substitutions in *Boechera *species**. Shaded red letters correspond to the sequence of the mature miRNA. Nucleotide substitutions of conserved miRNAs in other plant species compared with the corresponding miRNAs in *Boechera *species are shown as italicized, bold and underlined blue letters. MiRNA precursors could be slightly longer than the sequences shown in this figure.

Evaluation of the pre-miRNAs was also based on A+U content. The miRNA precursors have A+U content ranging from 41.96 to 63.46% (Table 1 &2; Figure 4, 5, 6 &7), similar to proportions observed in other plant species [41]. Consistent with general notion, the majority of identified *Boechera *miRNA precursors contain more A+U nucleotides than G+C [42,43]. It is also important to note that the formation of a stem-loop structure is not a unique feature of miRNAs, since other RNAs such as mRNA, rRNA, and tRNA can also form similar structures. For this reason, uniform systems for annotating new miRNAs comprising negative minimal fold energy (MFE), adjusted minimal fold energy (AMFE) and the minimal fold energy index (MFEI) have been developed [42-45] and have become generally accepted. Zhang et al [43] indicated that most identified miRNA precursors have an MFEI greater than 0.85, which is much higher than in tRNA (0.64), rRNA (0.59), or mRNA (0.65). However, a number of pre-miRNAs with lower MFEIs have been reported, provided the number of nucleotide substitutions in the particular conserved miRNA compared with other species does not exceed three (Table 1 &2; [41]).

### Microarray analysis of conserved miRNA families

The miRNAs identified from cDNA sequencing of floral tissues, using the bioinformatics described above, were further verified using a proprietary microarray analysis with LC Sciences, in order to validate their expression in sexual and apomictic *Boechera *flower tissues. The LC Sciences proprietary miRNA microarray chip that was used was designed by spotting all known plant miRNAs that were available in the miRBase Release 14 (total 1117 unique mature miRNAs) and the Plant miRNA Database, PMRD (total 5690 unique mature miRNAs). Subsequently hybridization was performed as described in Methods using isolated enriched *Boechera *small RNAs to confirm expressed conserved miRNAs. As expected, most (n = 50) mature miRNAs representing 22 miRNA families were identified to be conserved mainly compared to *A. thaliana*. The microarray assay confirmed 15 conserved families identified with the bioinformatics techniques. It is also noteworthy that 7 and 29 other miRNA families were respectively detected separately by the microarray and bioinformatics approaches (Additional file 2, Figure S2).

### *Boechera*-specific miRNA nucleotide substitutions (NSs) enhance pre-miRNA stem-loop stability

**Figure 8:**
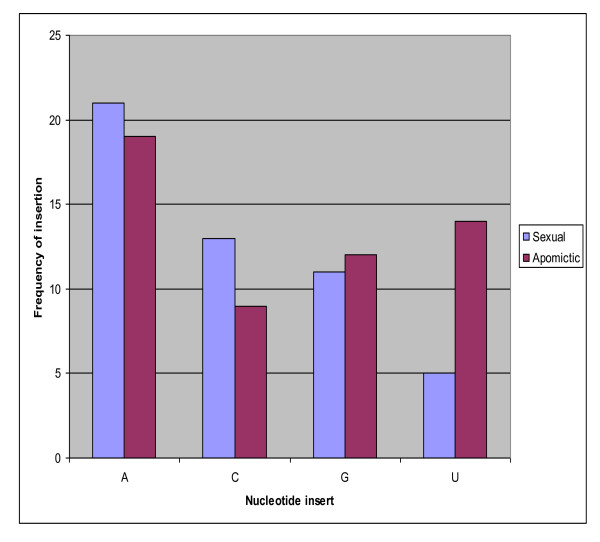
**Frequency of nucleotide substitutions related to *Arabidopsis thaliana *in mature *Boechera *miRNAs**.

The stability of a secondary structure is quantified as the amount of free energy released or used by forming base pairs. The more negative the free energy of a structure, the more likely is formation of that structure and its stability, because more stored energy is released, and this principle is used to predict the secondary structure of a particular sequence [46,47]. Out of the 30 stable *Boechera *pre-miRNA stem-loop structures obtained, 19 contain miRNAs with nucleotide substitutions (NSs) when compared with corresponding *Arabidopsis *or other plant miRNAs. The frequency of A, C and G substitutions were similar between sexual and apomictic mature miRNAs, while U appeared to show a higher rate of substitution in the apomictic mature miRNAs (Figure 8). Considering that a single nucleotide change in the sequence of a target site can affect miRNA regulation, NS could conceivably be under selection pressure to enhance the conformation and thermodynamic stability of the pre-miRNA stem-loop structure. We thus examined whether these *Boechera*-specific nucleotide changes had any effect on the structure and thermodynamic stability of their corresponding pre-miRNAs.

To do this, all pre-miRNAs containing miRNA NSs were selected from the various identified miRNA families (Figure 4, 5, 6 &7). For each pre-miRNA sequence, the Gibbs free energy (ΔG) was calculated using the mfold web server [48]. Importantly, the ΔG comparison was done between a pre-miRNA with the *Boechera*-specific miRNA sequence, and the same pre-miRNA with the miRNA sequence of (mostly) *Arabidopsis*, in other words "correcting" the NSs in the *Boechera *miRNA. In most cases, the *Boechera*-specific pre-miRNA showed significantly higher (p ≤ 0.05) thermodynamic stability (more negative free energy) than that of the pre-miRNA containing the "corrected" nucleotide substitution (Table 3). Similarly, when the corresponding pre-miRNAs in *Arabidopsis*/*Oryza *were "corrected" to *Boechera *miRNAs, most of the *Arabidopsis*/*Oryza *"new pre-miRNA" versions showed significantly lower (p ≤ 0.05) thermodynamic stability compared to the natural pre-miRNAs (Table 4).

**Table 3:** *Boechera *miRNA nucleotides substituted with those of *Arabidopsis*/*Oryza*.

Pre-miRNA	NSs	NN	ΔG	
	**Plant sp./*Boechera***		**Natural HP**	**"Corrected" HP**

***Sexual Species***				

bsex-MIR156a*	U/A	75	**-21.6**	**-19.3**
bsex-MIR157a	U/A	75	**-21.6**	**-19.3**
bsex-MIR396	U/G	143	-55.9	-56.8
bsex-MIR400*	A/G	76	**-12.0**	**-11.7**
bsex-MIR403*	G/C	75	**-18.7**	**-15.5**
bsex-MIR414* (1)	U/A, A/G	170	**-49.8**	**-48.2**
bsex-MIR414 (2)	U/A	221	-52.2	-56.4
bsex-MIR414 (3)	U/A	233	-57.9	-60.0
bsex-MIR852	U/G	89	-34.1	-34.7

***Apomictic Species***				
bapo-MIR156a*(1)	U/A	66	**-19.8**	**-15.6**
bapo-MIR156a*(2)	U/A	105	**-27.9**	**-26.7**
bapo-MIR157m*	A/G	119	**-38.2**	**-31.3**
bapo-MIR394a	C/A	126	-23.8	-26.2
bapo-MIR414* (1)	U/A, A/U	208	**-35.2**	**-34.2**
bapo-MIR414* (2)	U/A, A/G	170	**-49.8**	**-48.2**
bapo-MIR414* (3)	U/G	104	**-30.5**	**-29.2**
bapo-MIR415*	A/G, C/A	135	**-33.8**	**-30.3**
bapo-MIR861*	A/G, G/U	233	**-79.4**	**-71.4**

**HP**, Hairpin; **NN**, Number of nucleotides hairpin length; **NSs**, Nucleotide substitutions. Asterisk indicates cases where "correction" of *Boechera *miRNA NSs led to less stem-loop stability due to decrease in **Δ**G. Where there are two or more pre-miRNAs with the same miRNA, they are distinguished by numbers in brackets.

**Table 4:** Known plant miRNA nucleotides substituted with those of *Boechera*.

Pre-miRNA	Nucleotide Substitutions	NN	ΔG	
	**Plant sp./*Boechera***		**Natural HP**	**"Corrected" HP**

ath-MIR156a*	U/A	123	**-57.1**	**-52.4**
ath-MIR157m*	A/G	50	**-10.2**	**-9.3**
ath-MIR394*	C/A	117	**-53.1**	**-46.6**
osa-MIR396*	U/G	154	**-64.7**	**-60.1**
ath-MIR400*	A/U	102	**-38.4**	**-34.3**
ath-MIR403*	G/C	135	**-38.8**	**-35.4**

ath-MIR414	U/A, A/G	108	-22.0	-22.3
	U/G		**-22.0**	**-21.8**
	U/A, A/U		-22.0	-26.4
	U/A		-22.0	-23.3
	U/A, A/G, A/G		-22.0	-22.4

ath-MIR415*	A/G, C/A	110	**-27.0**	**-24.8**
ath-MIR852	U/G	202	-80.6	-80.8
ath-MIR861*	A/G, G/U	132	**-56.3**	**-51.6**

**HP**, Hairpin; **NN**, Number of nucleotides hairpin length; Asterisk indicates cases where "correction" of *Arabidopsis*/*Oryza *miRNA NSs led to less stem-loop stability.

In all, this nucleotide substitution-stability phenomenon was most common in our analyses of both apomictic (8 out of 9 miRNA families) and sexual *Boechera *(5 out of 9 miRNA families; Table 3), in addition to *Arabidopsis *(9 out of 11 miRNA families; Table 4). Naturally occurring miRNA NSs thus appear to confer optimal thermodynamic stability on pre-miRNA stem-loop structures in *Boechera*, and is consistent with similar analyses in other plants. For example, a similar comparison of the ΔG of predicted secondary structures of two variants of barley miR1137 precursor with a C and a G in the 13th position showed differences in stability between the variants [49]. Interestingly, Thakur et al [50] reported that species background may also be correlated with the calculation of both the minimum free energy and miRNA hairpin stability, although this difference appeared to be manifested at the level of mono- and dicots. Thus, at least with respect to the comparisons between closely related *Boechera *and *Arabidopsis *used here, our data imply that natural selection has guided sequence variation in these regulatory elements.

In one case pre-miRNA stability was also manifested on the intraspecific level, comparing sexual and apomictic *Boechera*. In the family miR394, the pre-miRNA of the sexual *Boechera *species has the same miRNA sequence as in *Arabidopsis*, however that of the apomictic species shows one C to A NS change at position seven (Figure 5A). The pre-miRNA stability was examined by introducing the apomictic NS into the sexual sequence at the same position and ΔGs compared. As expected there was a decrease in the negative ΔG by 6.5 kcal/mol in the "new" sexual pre-miRNA with the introduced apomictic NSs, suggesting that the sexual pre-miRNA is perhaps at its optimal thermodynamic stable state. This final evidence is consistent with *trans*-acting regulatory differences between sexual and apomictic ovules, the result of sequence variation in regulatory factors in the sexual (homozygous) versus apomictic (hybrid) genomes, as suggested by Sharbel et al [40].

### Conserved *Boechera *miRNAs target many transcription factors (TFs)

The BLAST analyses here have revealed many potential regulatory gene targets. Consistent with the results of functional studies in other plant species, such as *Arabidopsis*, rice and corn [26,51,52], the majority (40%) of target proteins in *Boechera *are transcription factors (Table 5; Additional file 3 Table S1). Transcription factors (TF) have been estimated in rice to be about 70% of conserved miRNA targets, while in wheat it has been predicted to be 35% [33,53]. The other targets are mostly associated with plant metabolism, development, signal transduction and response to environmental stress including cold, salinity, drought and nutritional deficiency [35,29,54,55].

**Table 5:** Transcription factor targets of conserved miRNA families in *Boechera *species.

miRNA family	Target protein	Function of target	Target gene (UPE)	E-value
miR156/157	Squamosa promoter binding protein like			
	SPL11	Transcription factor	AT1G27360 (11.430)	1
	SPL 2		AT5G43270 (11.987)	1
	SPL10		AT1G27370 (12.296)	1
	SPL15		AT3G57920 (14.449)	1
	SPL 9		AT2G42200 (16.239)	1
	SPL6		AT1G69170 (17.076)	1
				
miR159	Myb domain protein 120 (MYB120); DNA binding	Transcription factor	AT5G55020 (7.049)	3.5
miR160	Auxin Response Factor 10 (ARF10); transcription factor	Transcription factor	AT2G28350 (18.139)	1
miR167	Auxin response factor 8 (ARF8)	Transcription factor	AT5G37020 (17.281)	3.5
miR169	CCAAT-binding transcription factor (CBF-B/NF-YA) subunit B	Transcription factor	ATIG17590 (18.910)	2
MiR170/171	Scarecrow transcription factor family protein	Transcription factor	AT3G60630 (14.202)	1
miR172	RAP2.7, TOE1 | RAP2.7 (RELATED TO AP2.7) DNA binding	Transcription factor	AT2G28550 (16.639)	1.5
miR319	TCP10 (TCP Domain Protein 10)	Transcription factor	AT2G31070 (10.122)	3.5
	TCP4 (TCP family transcription factor 4)	Transcription factor	AT3G15030 (13.479)	3.5
				
miR396	AtGRF4 (Growth regulating factor 4)	Transcription activator	AT3G52910 (14.357)	2
miR408	TIL1 (TILTED 1); DNA binding/DNA-directed DNA polymerase/nucleic acid binding/nucleotide binding/zinc ion binding	Transcription factor	AT1G08260 (15.881)	4
miR414	WRKY DNA -binding domain	Transcription factor	AT4G31550	

(UPE**; Maximum energy to unpair the target site**, UPE range: **0.0-25.0**; E-value range: **0.0-4.0**)

The EST libraries from which the *Boechera *miRNAs were mined were flower-specific [39,40], and expectedly, a number of identified TF-targeting miRNAs have been associated with flower development in other species. For example, miR156 and miR157, the homologues of the squamosa-promoter binding proteins and whose function is well conserved across plant species [43], were identified in both apomictic and sexual *Boechera *(Table 5; Additional file 3, Table S1). In *Arabidopsis *these TF regulatory miRNAs have been reported to regulate the *Antirrhinum *floral meristem identity squamosa promoter binding protein-like (SPL) genes [56]. Other TF regulatory miRNA families which have regulatory roles during flower development in other species were also identified (Table 5; Additional file 3, Table S1), including miR156, miR159, miR164 and miR172, which have been implicated in the control of LFY expression, floral organ identity, and flowering time [27,57,58]. miR172 has furthermore been reported to regulate stem cell fate, and defines the inner boundary of the APETALA3 and PISTILLATA expression domains in *Arabidopsis *floral meristems [38].

A number of well-defined TF targeting miRNAs were also identified in *Boechera*. For example, miR160 and miR167 (Table 5; Additional file 3, Table S1) are associated with post-transcriptional regulation of the *A. thaliana *auxin response transcription factor (ARF) family genes [26,59]. miR319 is known to regulate the expression of TCP transcription factor genes whose down-regulations cause abnormalities in leaf development [16]. Vierstra [60] showed that miR394 regulates the messages of F-box proteins, which in turn target specific proteins for proteolysis by making them substrates for ubiquitination by SCF E3 ubiquitin ligases. Growth Regulating Factor genes, the targets of the miR396 family, are putative transcription factors that regulate cell expansion in leaf and cotyledon [61]. Argonaute, one of the important proteins in the regulation of miRNA biogenesis, is a target of miR403 whereas miR408 regulates a copper ion binding protein. The miR414 family regulates a number of other genes including the transcription factors, transducin family protein/WD-40 repeat family protein and peptidyl-prolyl cis-trans isomerase cyclophilin-type family protein.

### Expression patterns of transcription factor (TF) targets and apomixis in *Boechera*

The switch from sexual to apomictic seed production is hypothesized to involve global regulatory changes during ovule development which are induced by hybridization and/or polyploidy [9,62], both common characteristics of apomictic plants and parthenogenetic animals. Using data from a previously-published SuperSAGE analysis [39,40], the ovule expression patterns of putative target TFs for the miRNAs identified here were compared between sexual and apomictic *Boechera *across four ovule developmental stages. Of the 17 TFs identified as potential miRNA targets, expression data for 6 were found in the SuperSAGE libraries, including: the squamosa promoter binding protein like SPL6, SPL11 and SPL15, Myb domain protein 120 (MYB120), RAP2.7, TOE1 RAP2.7 (RELATED TO AP2.7) DNA binding and TCP10 (TCP family transcription factor 10), which are targets of the miRNA families miR156/157, miR159, miR172 and miR319 respectively.

**Figure 9:**
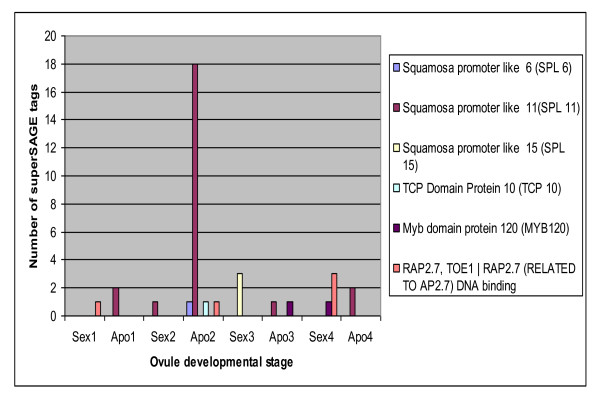
**Expression of transcription factors across *Boechera *sexual and apomictic ovule developmental stages**. Refer to Sharbel et al. [40] for descriptions of ovule stages.

**Figure 10:**
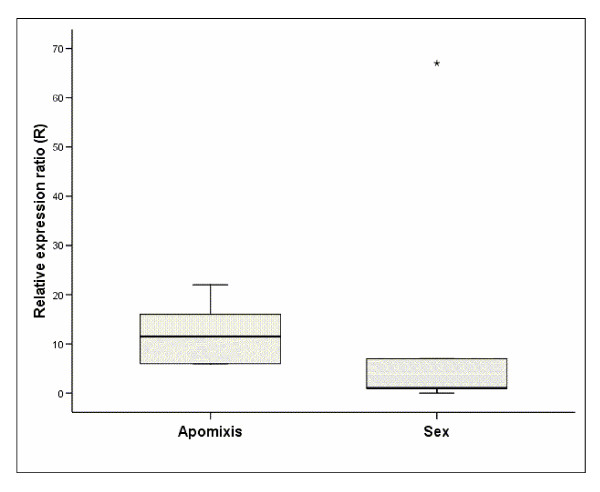
**Relative differential expression of SPL11 transcription factor in stage two of apomictic and sexual ovules**.

It is noteworthy that, whereas the other genes showed no significant differential expression levels between sexual and apomictic species, SPL11 was found to be significantly (p ≤ 0.05) up-regulated at the stage two of ovule development in apomictic species in all libraries studied (Figure 9). SPL11 also showed low level expression in all the other apomictic ovule stages and at only stage two of the sexual ovules. Using six apomictic and five sexual genotypes of *Boechera*, the differential expression of SPL11 at ovule stage two of floral development was further validated using quantitative Real Time-PCR. With the exception of a single sexual *B. divaricarpa *from Mule Ranch, Montana, all apomictic accessions clearly showed relatively higher expression of SPL11 than the sexuals (Figure 10), result which is consistent with the expression pattern observed with the SPL11 SuperSAGE tag (Figure 9). The single sexual outlier (Figure 10) for SLP11 implies that the expression pattern of this TF may not be a key factor associated with apomixis expression, but rather is associated with DNA sequence variation in regulatory factors in the hybrid *B. divaricarpa*. Alternatively, population-level variation for TF expression could be associated with the penetrance of the apomictic phenotype, which has been shown to be genotype-specific in *Boechera *[63].

## Conclusions

This study constitutes the first extensive insight into the conservation and expression of miRNAs in *Boechera *sexual and apomictic species. Of the expressed miRNA transcription factor targets observed, only the miR156/157 family target squamosa promoter binding protein-like 11 (SPL11) was found differentially expressed with significant (p ≤ 0.05) up-regulation at the stage two of ovule development in apomictic species. Also demonstrated here is that nucleotide changes in mature miRNAs significantly (p ≤ 0.05) enhance the thermodynamic stability of pre-miRNA stem-loops. This work will enhance subsequent elucidation of the repertoire of miRNA expression in *Boechera *towards revealing the potential role of miRNAs in the switch from sexual to apomictic reproduction.

## List of abbreviations

AGO, argonaute; ESTs, expressed sequence tags; GSS, genome survey sequences; miRNA, microRNA; miRISC, miRNA-induced silencing complex; MFE, minimum fold energy; MFEI, minimal fold energy index; AMFE, adjusted minimal fold energy; PMRD, plant miRNA database; TAIR, arabidopsis information resource; SBP, squamosa promoter binding protein; qRT-PCR, quantitative reverse transcription PCR.

## Methods

### Flower-specific *Boechera *454 cDNA libraries used

Floral cDNA libraries used in this study are those previously reported by Sharbel et al. [39,40]. These libraries were sequenced from pooled flower stages 1-12 [64] of three diploid sexual plants (Accessions ES910-2-2 K, 105.6-1 K and B07261) and three apomictic plants (Accessions 67.5-K, 300.6.1-1 K and 218.2-2 K).

### Conserved miRNA reference set for bioinformatics procedures

A total of 9274 previously reported non-redundant 21-24 nucleotides long miRNAs (including their precursor sequences) collected from 121 plant species were obtained from the Plant MicroRNAs Database (PMRD as of February 8, 2011; [65]). These miRNAs were defined as a reference set of miRNA sequences for the identification of potentially conserved miRNAs in *Boechera*. To avoid redundant miRNAs, duplicated miRNAs shared between different species within the database were removed. In all, 8433 non-redundant miRNAs were obtained, and these were used as query sequences for a BLASTn search against all original 454 sequence reads from the apomictic and sexual *Boechera *libraries.

### Identification of conserved miRNAs

The bioinformatics approaches used for identification of conserved miRNAs in *Boechera *species are outlined in Figure 1. The length of the EST sequences used to search for conserved miRNAs ranged between 51 and 478 nucleotides, with about 80% of them around 200 nucleotides long. In order to exclude all ESTs having exact matches to tRNA or rRNA sequences from further BLASTn searches, the sexual and apomictic EST libraries were first queried against ribosomal RNAs database from Rfam (http://www.sanger.ac.uk/Software/Rfam/) and the *Arabidopsis *transfer RNAs database (http://lowelab.ucsc.edu/GtRNAdb/Athal/). Rather than using the miRNA precursors for BLASTn searches against our databases, the analysis was based mainly on the mature miRNA sequences considering that only mature miRNAs are highly conserved in plants [42,43]. The following BLASTn parameters which gave the highest and most reliable number of hits (blastall -p blastn -m 8 -e 1 -W 7 -r 1 -q -1 -i) were used. All resulting EST sequences with an alignment length of 20-24 nucleotides, three or fewer mismatches and no gaps compared to previously identified plant miRNAs were selected and compared with each other to eliminate redundancies. The obtained non-redundant sequences were then used for the prediction of secondary structures and screening for miRNA precursor sequences. The secondary structures of pre-miRNAs were generated using the Mfold 3.2 software, which is based on Zuker folding algorithm principles [48].

The secondary structure of candidate pre-miRNA sequences were analysed and scored for their potential to form miRNA precursors. A stem-loop was selected as a candidate miRNA precursor if it satisfied most of the following generally accepted criteria: (1) the mature miRNA is 20-24nt with a maximum of three mismatches compared with the corresponding known miRNA in other plant species; (2) the miRNA precursor (pre-miRNA) sequence folds into a stable hairpin structure such that one arm of the hairpin contains the mature miRNA sequence; (3) the predicted secondary structure of the pre-miRNA has lower minimal free energy (MFE ≤-10 kcal/mol) and minimal free energy index (MFEI) than other types of RNA (e.g. tRNA, rRNA); (4) the predicted mature miRNA has an A+U content of 40-70%; and (5) no loop or gap in the mature miRNA sequences [41].

### Microarray validation of conserved plant miRNAs

The bioinformatically-identified miRNAs in floral tissues were further verified using a proprietary microarray analysis with LC Sciences, USA. The microarray assay was performed using 4 to 8 μg total RNA sample from pooled flower tissues of sexual and apomictic genotypes. The total RNA was size fractionated using a YM-100 Microcon centrifugal filter (Millipore) and the isolated small RNAs (< 300 nt) were 3'-extended with a poly(A) tail using poly(A) polymerase. An oligonucleotide tag was then ligated to the poly(A) tail for later fluorescent dye staining, and two different tags were used for two RNA samples in dual-sample experiments. Hybridization was performed overnight on a μParaflo microfluidic chip [spotted with all known plant mature miRNAs that were available in miRBase Release 14 (total 1117 unique mature miRNAs) and the Plant miRNA Database (total 5690 unique mature miRNAs)] using a micro-circulation pump (Atactic Technologies; [66]). On the microfluidic chip, each detection probe consisted of a chemically modified nucleotide coding segment complementary to a target miRNA, and a spacer segment of polyethylene glycol to extend the coding segment away from the substrate. The detection probes were made by *in situ *synthesis using PGR (photogenerated reagent) chemistry. The hybridization melting temperatures were balanced by chemical modifications of the detection probes, and hybridization was performed using 100 μL 6 × SSPE buffer (0.90 M NaCl, 60 mM Na_2_HPO_4_, 6 mM EDTA, pH 6.8) containing 25% formamide at 34°C. After RNA hybridization, tag-conjugating Cy3 and Cy5 dyes were circulated through the microfluidic chip for dye staining. The fluorescence data were collected on an Axon GenePix 4000B Microarray Scanner, and then analysed by first subtracting the background followed by normalization of the signals using a LOWESS filter (Locally-weighted Regression; [67]). A detectable miRNA on the array was identified if its signal intensity was higher than 3×(background standard deviation) and spot CV < 0.5, and p < 0.01 for the difference between Cy3 and Cy5 signals (LC Sciences).

### Prediction of *Boechera *gene targets of miRNA families

A BLASTn search (blastall -p blastn -m 8 -e 1 -W 7 -r 1 -q -1 -i) was employed to detect complementarity between the validated miRNAs and predicted target ESTs in sexual and apomictic *Boechera *(Additional file 3, Table S1). Putative miRNA targets were identified based on the total numbers of mismatched nucleotides between miRNAs and the alignment structures of potential targets. To identify potential regulatory targets, a BLAST search (blastall -p blastn -m 8 -e 1 -W 7 -r 1 -q -1 -i) was performed using the validated (from LC Sciences) conserved *Boechera *miRNAs against the *A. thaliana *protein-coding nucleotide databases (TAIR9 cDNA) using the miRU web server [68] from the *Arabidopsis *Information Resource (TAIR). The total number of allowed mismatches at complementary sites between miRNA sequences and potential mRNA targets in *Arabidopsis *were limited to a maximum of three, and no gaps were allowed at complementary sites. Finally, the *Boechera *homologues of potential targeted genes in *Arabidopsis *were chosen using a BLAST search (blastall -p blastn -m 8 -e 1 -W 7 -r 1 -q -1 -i) based on the degree of similarity of protein-coding mRNAs between *A. thaliana *and *Boechera*.

### Expression analysis of Transcription factor (TF) targets using SuperSAGE tags

Finally, a comparative gene expression analysis of TF targets from 11 miRNA families was carried out. First, a BLASTn search using TF genes from *Arabidopsis *against the assembled *Boechera *EST database was performed (blastall -p blastn -m 8 -e 1 -W 7 -r 1 -q -1 -i). For each Arabidopsis TF, homologous *Boechera *TFs with an alignment having a bit score ≥100 were selected. Next, 100% sequence matches between the *Boechera *TFs and expression tags from 8 ovule-specific *Boechera *SuperSAGE libraries [40] were found using a BLASTn search. Finally, the expression patterns of the selected *Boechera *TFs corresponding to the obtained SuperSAGE tags were compared across four different ovule developmental stages between a sexual and apomictic *Boechera *genotype [40].

### Quantitative RT-PCR validation of differential SPL11 expression in ovule stage two of *Boechera *flowers

Six accessions of apomictic and five of sexual *Boechera *were selected for the validation the differential expression of SPL11 (Additional file 4, Table S2). From these accessions, stage two ovules were micro-dissected, RNAs isolated and cDNAs prepared as described in Sharbel et al. [40]. The forward primer 5'-CAAAGTGCCCAAAAGTTACCGTGAGT-3' and reverse primer 5'-ACGCCTCGCATTATGATGAGAAAGA-3' with amplicon size of 137 nucleotides long were used for the qRT-PCR. Primers were designed avoiding intronic regions (to ensure the elimination of likely DNA contamination in samples) using the following parameters: temperature; 60°C, 20% < CG content < 80%, and PCR product size < 150 bp. For the real-time PCR reactions, the SYBR Green PCR Master Mix (Applied Biosystems) was used. qRT-PCR amplifications were performed in a 7900 HT Fast RT-PCR system (Applied Biosystems) with the following temperature profile for SYBRgreen assays: initial denaturation at 90°C for 10 min, followed by 40 cycles of 95°C for 15 s, and 60°C for 1 min. The Ct, defined as the PCR cycle at which a statistically significant increase of reporter fluorescence is first detected, was used as a measure for the starting copy numbers of the target gene. The mean expression level and standard deviation for each set of three technical replicates for each cDNA was calculated. Relative quantitation and normalization of the amplified targets were performed by the comparative ΔΔCt method in reference to the expression levels of the housekeeping gene ubiquitin [69].

## Authors' contributions

SA: Project development, bioinformatics, microarray analysis and draft of manuscript. JMC: Project development, analytical supervision and sample preparation. HV: Project development and sample preparation. TFS: Project development, bioinformatics and draft of manuscript. All authors have read and approved the final manuscript.

## Supplementary Material

Additional file 1 ***Boechera *stem-loop structures**. List of predicted pre-miRNA structures of conserved miRNAs identified in *Boechera *species. Click here for file

Additional file 2 ***Boechera *miRNA families**. Grouping of miRNA families identified by bioinformatics and microarray assay. Click here for file

Additional file 3 **Predicted miRNA targets**. Gene targets of conserved miRNA families in *Boechera *species. Click here for file

Additional file 4 ***Boechera *genotypes**. *Boechera *genotypes used for qRT-PCR validation of differential SPL11 expression. Click here for file
