# Supplementary material for: Analysis of conserved microRNAs in floral tissues of sexual and apomictic Boechera species
Source: BMC Genomics. 2011 Oct 11;12:500. doi: 10.1186/1471-2164-12-500 (PMC3208272; doi:10.1186/1471-2164-12-500)
Supplement: Additional file 1 — Boechera stem-loop structures. List of predicted pre-miRNA structures of conserved miRNAs identified in Boechera species. [file 1471-2164-12-500-S1.DOC]

**Additional file 1, Figure S1: List of predicted pre-miRNA structures of conserved miRNAs identified in *Boechera* species**

MiR156 family

**Sexual ath156a**

Initial dG = -21.60

10 20 30

| U**UGACAG** **AG** - UUU CG

UGUGGAU **AAG** **AGAGAGC** **ACA**G GAGU \

AUACCUG UUU UCUCUCG UGUU CUUA C

^ UGA---- GA A CC- CC

70 60 50

**Apomictic Ath156a**

Initial dG = -26.50

10 20 30 40 50

GA| **UGAC** **AA**- **AG** **C**AAUCAGAUUCAU - AA

CGAGGAGGAGGA **AG** **GAGAG** **AGCA** GGAUG C \

GUUCUUCUUCUU UC CUCUU UCGU CUUAC G C

C-^ ---- GAG G- AGCAACACACUCU U CU

100 90 80 70 60

**Apomictic Ath156a**

Initial dG = -19.80

10 20 30

U**U** **G**- **AG** -| UUU CG

**GACA**  **AAG** **AGAGAGC** **AC**AG GAGU \

CUGU UUU UCUCUCG UGUU CUUA C

AC GA GA A^ CC- CC

60 50 40

MiR157 family

**Sexual ath157m**

Initial dG = -21.60

10 20 30

| **UUGACAG** **AG** - UUU CG

UGUGGAU **AAG** **AGAGAGC** **ACA**G GAGU \

AUACCUG UUU UCUCUCG UGUU CUUA C

^ UGA---- GA A CC- CC

70 60 50

**Apomictic ath157m**

Initial dG = -38.20

10 20 30 40 50 60

-| U U A **UGA** **A** - CAUCAA GAGAA C

GAAACC CA GA GUUGA U**U** **CAG** **AGAGAGAGGG** **CAC** GAUCUU GCAC C

UUUUGG GU CU CGACU AA GUC UUUCUUUUCC GUG UUAGAG UGUG A

C^ U - C UAA A A ------ AAG-- U

110 100 90 80 70

MiR160 family

**Apomictic ath160a**

Initial dG = -46.30

10 20 30 40 50

| AUA **C**  **CC**- A AUAAC AGU

AAGGAUUU GUCG**UGC** **UGGCU** **CUGUAUGCCA**C AG AUCGAUUU \

UUUCUGAA CAGUACG ACUGA GACAUGCGGUG UC UAGCUAAA U

^ AC- A UGA A AC--- ACA

. 100 90 80 70 60

MiR161 family

**Sexual ath 161a**

Initial dG = -22.70

10 20 30 40

**GAAA** **C** **AUC**----| U UUU A C AUC

GC**U** **GUGA** **UAC** **GGGGU** GAU CCUCUC AU AU A

CGA CAUU AUG CCCCA CUA GGAGAG UG UG C

AAA- A CCUGCUA^ - C-- C A AAU

90 80 70 60 50

MiR162 family

**Apomictic ptc162a**

Initial dG = -40.70

10 20 30 40

| G C C C GGAAACAA

CGCUGGA GCAG GGUU AUCGAUCU UUCGUG A

GU**GACCU** **CGUC** **CCAA** **UAGCU**AGA AAGUAC A

^ **A** **U**  **A** U AAAAAAAU

80 70 60 50

MiR167 family

**Sexual ath167a**

Initial dG = -48.20

10 20 30 40

**U** **C** UA U - ---| UG U

**GAAGCUGC** **AGCAUGAUCUA**AU GC UUC UUUCU CCGUUGU U U

CUUUGACG UUGUACUAGAUUA UG GAG AGAGA GGUAGCA A U

A C GC U U AUU^ GU C

. 90 80 70 60 50

**Apomictic Ath167a**

Initial dG = -48.20

10 20 30 40

**U** **C** UA U - ---| UG U

**GAAGCUGC** **AGCAUGAUCUA**AU GC UUC UUUCU CCGUUGU U U

CUUUGACG UUGUACUAGAUUA UG GAG AGAGA GGUAGCA A U

A C GC U U AUU^ GU C

. 90 80 70 60 50

MiR170 family

**Apomictic ath170**

Initial dG = -49.00

10 20 30 40 50 60

| CC UU C C CU CU CCCCCACCACAC

ACGAGAGAGU CU GAUAUUGGC UGGUUCA UCAGAU UC GA G

UGCUCUCUCG GA **CUAUAACCG** **GCCGAGU** **AGU**CUA AG CU U

^ U- CU **C** **U**  UU AU CUUACACAUACA

. 110 100 90 80 70

MiR172 family

**Apomictic ath172a**

Initial dG = -31.80

10 20 30 40 50

G UA GAU GGAUG .-AUAGAGA G .-A| GA

CU AGAUGCG AUCA GGGU AC CC GGCUG UUCA U

GA UU**UACGU** **UAGU** **CCUA** UG GG UCGAU AAGU U

G **CG** **AGU** **AGA**G- \ ------- G \ -^ AG

140 130 120 60

MiR319 family

**Apomictic ath319a**

Initial dG = -83.20

10 20 30 40 50 60 70 80 90

| A U GAAA A GG G AC C UC AA C AG AAG

GAAGGAG UUCUUUCAGUCCAG CAUGGAUA AAGAAG G UAGAAAUAUCU CCG UCAU CA CA CACU GUGGU AAGA A

C**UUCCUC** **AGGGAAGUCAGGUU** GUGUCUAU UUCUUC C GUUUUUAUAGA GGC AGUA GU GU GUGA CGCCA UUUU G

^ **G** U ---- - UA G GU A GU CA - AA AGA

180 170 160 150 140 130 120 110 100

MiR394 family

**Sexual ath 394a**

Initial dG = -53.90

10 20 30 40 50

GA - **UUC** UCU--| U U AGC

GACAGA UCU **UUGGCA** **UGUCCACCUCC**UCUC AUAUA AUG GUAUA G

UUGUCU AGA AACCGU ACGGGUGGAGGAGAG UGUGU UGC UAUAU U

AG U CAU UGUUU^ - U GCA

110 100 90 80 70 60

**Apomictic ath394a**

Initial dG = -24.10

10 20 30 40

C- CCUCAAG- AU AU .-ACCU| GC

CUGU UGGA GACA AAU CAGACA UUGUU \

GACA ACCU **CUGU** **UUA** **GUU**UGU AACAG C

AC U**CCUCCAA** **C**- **CG** \ ----^ AU

120 110 100 50

MiR396 family

**Sexual ghr396**

Initial dG = -56.30

10 20 30 40 50 60 70

| U CAUU C U GA **CCAC** **UU** **UGAACGG** - - CA GC

GGACG GGCGGUAG GA GGUGG GG GUU**U** **AGGC** **UCU** UUGC GGCCACG GUG UGUG \

CCUGC CUGCCAUU CU UCACC CC CAAG UCUG AGA AGCG CCGGUGU CAU ACGC G

^ - C--- U U UA UUC- C- ------- A U A- UC

140 130 120 110 100 90 80

MiR400 family

**Sexual ath400**

Initial dG = -12.00

10 20 30

GUUCCA AU--------- CC-| GACU CAA

UCCC CAUGUA CUCUUA UG \

AGGG **GUAUAU** **GAGAGU** AC A

C----- CUUACAU**CACU** **UAU**^ **AU**UU CUA

70 60 50 40

MiR403 family

**Sexual ath403**

Initial dG = -18.70

10 20 30

C| AA AA**UUA** **UC** **CAAA**-- - UA

GAA ACCC **GAU** **ACGCA** **CUCC**UUU C \

CUU UGGG CUA UGCGU GAGGAAA G C

-^ AC ----- U- CAAAAC A AC

70 60 50

MiR408 family

**Sexual ath408**

Initial dG = -49.70

10 20 30 40 50 60 70

A A C UG AU U ------| CAA A AUU UUU U UAAA

GAAG AGA AAAG GUA GAGA AGA CAGGGAA GCAG GCAUGG GAG AC AAAACAU \

UUUC UCU UUUC UAU CUCU UCU **GUCCCUU** **CGUC** **CGUA**CC CUC UG UUUUGUG C

- C C UU CU U CCAU**CG**^ **CUC** **A**  CAU --- - UCAG

140 130 120 110 100 90 80

**Apomictic ath408**

Initial dG = -35.20

10 20 30 40

GA| CAA G AUU UUU U UAAA

CAGGGAA GCAG GCAUGG GAG AC AAAACAU \

**GUCCCUU** **CGUC** **CGUA**CC CUC UG UUUUGUG C

**CG**^ **CUC** **A** CAU --- - UCAG

80 70 60 50

MiR414 family

A. **Apomictic ath414**

**Apomictic ath414**

Initial dG = -35.20

10 20 30 40 50 60 70

UU A AC U UUUU UCUUU U UCGACUAACACCU .-U| UU

GUU GAGAUGA GA AG ACG UGGU UGAUGGU AC GUGAC GGU \

CAA UUCUACU CU **UC** **UGC** **ACUA** **ACUACU**A UG UACUG CCA C

U- G -- - **UACU** **CU**--- C CUUCUUCUCCUUC \ -^ UA

200 190 180 170 160

**Apomictic ath414**

Initial dG = -49.80

10 20 30 40 50 60 70 80

**UCA** **A** **A** **A** **AU**- **U** UA GUGAAGC- UUAG -- UC- ---- --| UU

**UC** **UC** **UC** **UC** **CG** **CG**UCGUCAUCAU UUGGUCU CAUUACU CAA GUC UGAGC AGCG GCU \

GG AG AG AG GC GCAGUAGUGGUG AGCUAGA GUGAUGA GUU CAG GCUCG UUGC CGA A

CGA A A - GCC - CG AGAAGGCA UACA UG UUU AGUU AG^ CG

. 160 150 140 130 120 110 100 90

**Apomictic ath414**

Initial dG = -30.50

10 20 30 40 50

**UCAUC**| **GUC** C U U C CGAG-- CGA

**GUCAUCAUCAUC** **A**U AUCUUCA CU CCU CU UGA U

UAGUAGUAGUAG UA UAGAGGU GA GGA GA ACU G

UUGUU^ ACA A U U A AAUUUA UAA

100 90 80 70 60

B. **Sexual ath414**

Initial dG = -52.20

10 20 30 40 50 60 70 80 90 100 110

GU| UAGG A GAUU UGCG GAU U AAGGCG-- AGGG AUU GGGGAAG G G UGGU - UA---- C AU

UC AG AUGGA UGGCG GA UGAUG UGA UGGAGA GAG UG AUU AAU GUC ACUGU GCU CGGUUC UAAUGG \

AG UC UACCU **ACUGC** **CU** **ACUAC** **ACU** AUCUCU CUU AC UAG UUA UAG UGACA CGA GUUAAG GUUGCC C

C-^ UA-- G ---- **UA**-- **ACU** **U**  AAUAUUAA GCAA GU- AUACA-- - G UU-- U CAGUUC U UC

220 210 200 190 180 170 160 150 140 130 120

**Sexual ath414**

Initial dG = -57.40

10 20 30 40 50 60 70 80 90

AAAGC UA UCUAG GA GA UGGUGUG .-UGAU AA UGGA-- A A GG- | G

GGA UGG GUGGUGGU GA AUG GAUU CGGAGAU GUUG AGGCG GA GGGG GAUUU GGGA--AGAUU A

CCU **ACU** **UACUACUA**  **CU** **UAC** CUAA GUCUCUG CGAC UUCGU UU CCUC CUAAG UCCU UCUGG A

----- **GC** ----- **AC** **U**A UAUUAA- \ ---- GG UAAGUG A - UAA \ ^ U

230 220 210 200 150 140 130 100

**Sexual ath414**

Initial dG = -49.80

10 20 30 40 50 60 70 80

**UCA** **A** **A** **A** **AU**- **U** UA GUGAAGC- UUAG -- UC- ---- --| UU

**UC** **UC** **UC** **UC** **CG** **CG**UCGUCAUCAU UUGGUCU CAUUACU CAA GUC UGAGC AGCG GCU \

GG AG AG AG GC GCAGUAGUGGUG AGCUAGA GUGAUGA GUU CAG GCUCG UUGC CGA A

CGA A A - GCC - CG AGAAGGCA UACA UG UUU AGUU AG^ CG

. 160 150 140 130 120 110 100 90

MiR415 family

**Apomictic ath415**

Initial dG = -33.70

10 20 30 40 50 60

GC- GU- --- **A** **A** **A** .-**U**CCU| UCCAA UU UGUG AUG U

CC U**GA** **CAG** **GAAGA** **ACAGA** **CA** GGU AUU CCUU CGG GA U

GG ACU GUC CUUCU UGUCU GU CCA UAA GGAA GCC UU C

GCA AGU AUA C A A \ ----^ UG--- GG UUA- GAA G

130 120 110 80 70

MiR852 family

**Sexual ath852**

Initial dG = -34.10

10 20 30 40

U| U **AGC** **G** - AA-- C

GGAUAUA CAGAG**AAGAUA** **GCCUUA** **GU CUG** GAUAAG \

CCUAUAU GUUUCUUCUAU UGGAGU CG GAU CUAUUC U

A^ U --- A U AUAC C

80 70 60 50

MiR861 family

**Apomictic ath861-5p**

Initial dG = -74.40

10 20 30 40 50 60

- - **U** **UCA** UU U C UGU| AGA .-UC AUA

**CCUUGGAGA** **AAUG** **GCU** **A**GAUCC GUCAG GA UCU AGAU CCAG AC \

GGGGCCUUU UUGC CGA UCUAGG CAGUU CU AGA UCUA GGUC UG G

U C U --- UC - - ---^ --- \ -- AGA

230 220 210 200 190
