# Supplementary material for: Analysis of conserved microRNAs in floral tissues of sexual and apomictic Boechera species
Source: BMC Genomics. 2011 Oct 11;12:500. doi: 10.1186/1471-2164-12-500 (PMC3208272; doi:10.1186/1471-2164-12-500)
Supplement: Additional file 2 — Boechera miRNA families. Grouping of miRNA families identified by bioinformatics and microarray assay. [file 1471-2164-12-500-S2.DOC]

**Additional file 2, Figure S2: Grouping of miRNA families identified by bioinformatics and microarray assay**

miR162

miR319

miR394

miR396

miR398

miR400

miR403

miR414

miR415

miR444

miR472

miR482

miR776

miR820

miR824

miR835

miR838

miR840

miR841

miR845

miR846

miR852

miR854

miR857

miR859

miR860

miR861

miR865

miR869

miR156

miR157

miR159

miR160

miR161

miR164

miR166

miR167

miR169

miR170

miR172

miR395

miR399

miR408

miR529

***Microarray assay***

***Bioinformatics***

miR158

miR163

miR165

miR168

miR482

miR530

miR535
