# Supplementary material for: Analysis of conserved microRNAs in floral tissues of sexual and apomictic Boechera species
Source: BMC Genomics. 2011 Oct 11;12:500. doi: 10.1186/1471-2164-12-500 (PMC3208272; doi:10.1186/1471-2164-12-500)
Supplement: Additional file 3 — Predicted miRNA targets. Gene targets of conserved miRNA families in Boechera species. [file 1471-2164-12-500-S3.DOC]

**Additional file 3, Table S1: Gene targets of conserved miRNA families in *Boechera* species**

| **miRNA family** | **Target protein** | **Function of target** | **Target gene (UPE)** | **E-value** |
| --- | --- | --- | --- | --- |
| miR156/157 | Squamosa promoter binding protein like SPL11  SPL 2  SPL10  SPL15  SPL 9  SPL6 | Transcription factor | AT1G27360 (11.430)  AT5G43270 (11.987)  AT1G27370 (12.296)  AT3G57920 (14.449)  AT2G42200 (16.239)  AT1G69170 (17.076) | 1.0  1.0  1.0  1.0  1.0  1.0 |
| miR159 | Myb domain protein 120 (MYB120); DNA binding | Transcription factor | AT5G55020 (7.049) | 3.5 |
| miR160 | Auxin Response Factor 10 (ARF10); transcription factor | Transcription factor | AT2G28350 (18.139) | 1.0 |
| miR161 | Pentatricopeptide (PPR) repeat-containing protein |  | AT5G41170 (15.968) | 1.0 |
| miR162 | Dicer-like1 (DCL1); ATP-dependent helicase/ ribonuclease III | miRNA biogenesis | AT1G01040 (20.324) | 2.0 |
| miR166 | F box family protein |  | AT1G66310 (20.850) | 3.5 |
| miR167 | Auxin response factor 8 (ARF8) | Transcription factor | AT5G37020 (17.281) | 3.5 |
| miR169 | CCAAT-binding transcription factor (CBF-B/NF-YA) subunit B | Transcription factor | ATIG54160 (18.910) | 2.0 |
| miR170/171 | Scarecrow transcription factor family protein | Transcription factor | AT3G60630 (14.202) | 1.0 |
| miR172 | RAP2.7, TOE1 | RAP2.7 (RELATED TO AP2.7) DNA binding | Transcription factor | AT2G28550 (16.639) | 1.5 |
| miR319 | FMN binding  TCP10 (TCP Domain Protein 10)  TCP4 (TCP family transcription factor 4)  PCNA2 (Proliferating Cell Nuclear Antigen 2)  AtMYB104 (myb domain protein 104) | Transcription factor  Transcription factor  Transcription factor  Transcription factor | AT3G21140 (7.887)  AT2G31070 (10.122)  AT3G15030 (13.479)  AT2G29570 (15.100)  AT2G26950 (18.403) | 3.5  3.5  3.5  3.5  2.5 |
| miR394 | F-box family protein  GDSL-motif lipase/hydrolase family protein  Zinc finger (AN1-like) family protein |  | AT1G27340 (14.870)  AT3G48460 (15.922)  AT4G22820 (19.956) | 1.0  3.0  3.5 |
| miR396 | AtGRF4 (Growth regulating factor 4)  FLU (Fluorescent in blue light)  Pentatricopeptide (PPR) repeat-containing protein | Transcription activator  Binding | AT3G52910 (14.357)  AT3G14110 (16.108)  AT2G15630 (16.675) | 2.0  2.5  3.5 |
| miR398 | Transposable element gene  CSD1 (Copper/Zinc Superoxide Dismutase 1) |  | AT2G10910 (12.002)  AT1G08830 (15.749) | 3.5  3.5 |
| miR399 | PHO2/UBC24 (Phosphate 2); ubiquitin-protein ligase  CYP705A30; electron carrier/ heme binding / iron ion binding / monooxygenase/ oxygen binding | Metabolism | AT2G33770 (11.851)  AT3G20940 (13.669) | 0.5  3.0 |
| miR400 | SCRL15 (SCR-Like 15)  Pentatricopeptide (PPR) repeat-containing protein |  | AT2G05335 (9.858)  AT4G19440 (13.522) | 3.5  2.0 |
| miR403 | WD-40 repeat family protein / beige-related  Pentatricopeptide (PPR) repeat-containing protein  Leucine-rich repeat family protein  Protein binding / tubulin-tyrosine ligase  Methyltransferase family protein  Sodium/calcium exchanger family protein / calcium-binding EF hand family protein  F-box family protein |  | AT1G03060 (13.238)  AT4G01030 (13.992)  AT1G13910 (14.118)  AT1G77550 (15.764)  AT3G17310 (15.945)  AT1G53210 (16.920)  AT3G49040 (18.676) | 3.0  3.5  3.5  3.0  3.5  3.5  3.5 |
| miR408 | TIL1 (TILTED 1); DNA binding / DNA-directed DNA polymerase/ nucleic acid binding / nucleotide binding / zinc ion binding |  | AT1G08260 (15.881) | 4.0 |
| miR414 | NAP1;1 (Nucleosome Assembly Protein1;1)  Glutaredoxin family protein  Transducin family protein / WD-40 repeat family protein  WRKY DNA -binding domain  Peptidyl-prolyl cis-trans isomerase cyclophilin-type family protein  B3 family protein | DNA binding  Transcription factor  Transcription factor | AT4G26110 (3.328)  AT3G28850 (8.503)  AT4G38480 (8.703)  AT3G01080 (10.157)  AT4G33060 (10.819)  AT3G53310 (11.197) | 0.5  1.0  1.0  0.5  0.5  0.5 |
| miR415 | Leucine-rich repeat family protein / protein kinase family protein  Oxidoreductase, 2OG-Fe(II) oxygenase family protein |  | AT1G04210.1(15.856)  AT4G02940.1(11.345) | 0.0  1.5 |
| miR472 | Disease resistance protein (CC-NBS-LRR class), putative  ATP binding / protein binding |  | AT5G63020.1(18.777)  AT1G61310.1(17.510) | 0.5  1.0 |
| miR482 | Disease resistance protein (CC-NBS-LRR class), putative |  | AT1G63360.1(16.404) | 0.0 |
| miR529 | Transcriptional activator, putative  Forkhead-associated domain-containing protein / FHA domain-containing protein  ABC transporter family protein | Transcription factor | AT3G07220.1(12.418)  AT3G07260.1(17.655)  AT5G19410.1(14.602) | 1.5  1.5  2.0 |
| miR776 | CCR4-NOT transcription complex protein, putative  Crooked neck protein, putative / cell cycle protein, putative  Dof-type zinc finger domain-containing protein | Transcription factor | AT3G44240.1(18.460)  AT3G51110.1(14.099)  AT2G28510.1(12.306) | 1.0  2.0  2.5 |
| miR820 | DNAJ heat shock family protein |  | AT2G20560.1(23.049) | 2.0 |
| miR824 | AGL16 (AGAMOUS-LIKE 16); transcription factor | Transcription factor | AT3G57230.1(12.902) | 0.0 |
| miR835 | AMK2 (Adenosine monophosphate kinase); ATP binding / adenylate kinase/ nucleobase, nucleoside, nucleotide kinase/ nucleotide kinase/ phosphotransferase, phosphate group as acceptor |  | AT5G47840.1(12.099) | 1.5 |
| miR838 | Vacuolar calcium-binding protein-related  Myb family transcription factor | Transcription factor | AT1G62480.1(5.416)  AT2G13960.1(12.727) | 1.0  1.0 |
| miR841 | HTA8 (HISTONE H2A 8); DNA binding |  | AT2G38810.1(22.435) | 0.0 |
| miR845 | Transposable element gene  DNA-directed DNA polymerase family protein |  | AT5G35756.1(23.809)  AT4G32700.1(17.375) | 1.0  2.5 |
| miR846 | Jacalin lectin family protein |  | AT1G52050.1(15.286) | 0.0 |
| miR854 | AGO7 (ARGONAUTE7); nucleic acid binding  Ribosomal protein S15 family protein  TEL2 (TERMINAL EAR1-LIKE 2); RNA binding / nucleic acid binding / nucleotide binding  Histone H1.2 |  | AT1G69440.1(0.138)  AT1G80620.1(0.977)  AT1G67770.1(3.170)  AT2G30620.1(7.106) | 1.0  1.0  1.0  1.0 |
| miR857 | Transposable element gene |  | AT5G31821.1(12.318) | 2.5 |
| miR859 | F-box family protein |  | AT3G17265.1(16.456) | 0.0 |
| miR860 | Transposable element gene |  | AT3G33139.1(13.696) | 2.5 |
| miR861 | Nucleic acid binding / nucleotide binding |  | AT4G35785.1(15.634) | 1.5 |
| miR865 | IMB1 (IMBIBITION-INDUCIBLE 1); DNA binding  MCM9; ATP binding / DNA binding / DNA-dependent ATPase/ nucleoside-triphosphatase/ nucleotide binding  RPL4; poly(U) binding / structural constituent of ribosome |  | AT2G34900.1(19.466)  AT2G14050.1(19.277)  AT1G07320.1(19.794) | 0.0  1.5  1.5 |
| miR869 | Encodes a Plant thionin family protein |  | AT3G48205.1(17.114) | 1.5 |
