# Supplementary material for: Analysis of conserved microRNAs in floral tissues of sexual and apomictic Boechera species
Source: BMC Genomics. 2011 Oct 11;12:500. doi: 10.1186/1471-2164-12-500 (PMC3208272; doi:10.1186/1471-2164-12-500)
Supplement: Additional file 4 — Boechera genotypes. Boechera genotypes used for qRT-PCR validation of differential SPL11 expression. [file 1471-2164-12-500-S4.DOC]

**Additional file 4, Table S2: *Boechera* genotypes used for qRT-PCR validation of differential SPL11 expression**

| **Accession** | **Reproduction** | **Ploidy** | **Taxon** | **Geographic origin** |
| --- | --- | --- | --- | --- |
| B08-02 | Apo | 2 | *B. polyantha* | Birch Creek, Ravalli Co, Montana |
| B08-81 | Apo | 2 | *B. divaricarpa* | Vipond Park, Montana |
| B08-43 | Apo | 3 | *B. polyantha X retrofracta* | Highwood Mtns, Montana |
| B08-66 | Apo | 3 | *B. polyantha X retrofracta* | Highwood Mtns, Montana |
| B08-104 | Apo | 3 | *B. divaricarpa* | Lost Trail Meadow, Idaho |
| B08-215 | Apo | 3 | *B. divaricarpa* | Carson Pass, California |
| B08-369 | Sex | 2 | *B. divaricarpa* | Mule Ranch, Montana |
| B08-355 | Sex | 2 | *B. stricta* | Gold Creek, Colorado |
| B08-329 | Sex | 2 | *B. polyantha* | Big Hole Pass, Beaverhead Co, Montana |
| B08-344 | Sex | 2 | *B. polyantha X ?* | Bandy Ranch, MT |
| B08-390 | Sex | 2 | *B. holboellii* | Panther Creek, Idaho |
